# Giant magnetoelectric effect at the graphone/ferroelectric interface

**DOI:** 10.1038/s41598-018-30010-x

**Published:** 2018-08-20

**Authors:** Jie Wang, Yajun Zhang, M. P. K. Sahoo, Takahiro Shimada, Takayuki Kitamura, Philippe Ghosez, Tong-Yi Zhang

**Affiliations:** 1 Department of Engineering Mechanics & Key Laboratory of Soft Machines and Smart Devices of Zhejiang Province, Zhejiang University, 38 Zheda Road, Hangzhou, 310007 China; 2 Theoretical Materials Physics, Q-MAT, CESAM, University of Liège, B-4000 Liège, Belgium; 3 Department of Mechanical Engineering and Science, Kyoto University, Nishikyo-ku, Kyoto, 615-8540 Japan; 4 Shanghai University Materials Genome Institute and Shanghai Materials Genome Institute, Shanghai University, 99 Shangda Road, Shanghai, 200444 China

## Abstract

Multiferroic heterostructures combining ferromagnetic and ferroelectric layers are promising for applications in novel spintronic devices, such as memories with electrical writing and magnetic reading, assuming their magnetoelectric coupling (MEC) is strong enough. For conventional magnetic metal/ferroelectric heterostructures, however, the change of interfacial magnetic moment upon reversal of the electric polarization is often very weak. Here, by using first principles calculations, we demonstrate a new pathway towards a strong MEC at the interface between the semi-hydrogenated graphene (also called graphone) and ferroelectric PbTiO_3_. By reversing the polarization of PbTiO_3_, the magnetization of graphone can be electrically switched on and off through the change of carbon-oxygen bonding at the interface. Furthermore, a ferroelectric polarization can be preserved down to ultrathin PbTiO_3_ layers less than one nanometer due to an enhancement of the polarization at the interface. The predicted strong magnetoelectric effect in the ultimately thin graphone/ferroelectric layers opens a new opportunity for the electric control of magnetism in high-density devices.

## Introduction

The coupling of ferromagnetic and ferroelectric orders in multiferroic materials has attracted continuous attention due to its potential use in novel spintronic devices such as data-storage devices for writing electrically and reading magnetically^1–5^. In perovskites, ferroelectricity typically requires a formal *d*^0^ electron configuration that drives electric polarization through cation off-centering, whereas a partially filled *d* state is required for a magnetic moment^6^. As a result, the coexistence of ferromagnetic and ferroelectric orders is relatively rare in single-phase compounds. By contrast, composite multiferroics made of ferroelectric and ferromagnetic constituents are abundant in the form of heterostructures, in which MEC exists at the interface through different mechanisms^7–12^. One kind of such heterostructures investigated so far consist in multilayers combining a conventional ferromagnetic metal and a ferroelectric insulator perovskite oxide. For example, the interface between ferroelectric BaTiO_3_ (BTO) and ferromagnetic Fe or Co has been intensively investigated due to the presence of interfacial MEC^13–15^. The interfacial MEC is attributed to the change of magnetic moments either at the interfacial atoms^13,14^ or at the interfacial oxidized Fe layer^15^, whose magnetization can be tuned on reversal of the ferroelectric polarization. Nevertheless, the change of interfacial magnetization upon reversal of the electric polarization in the conventional Fe/BaTiO_3_ system^13,14^ is still small. Practical application of MEC in memory devices requires new magnetoelectric heterostructures that have strong magnetoelectric effect.

Moreover, a practical tendency towards ultrahigh-density memory devices drives the current research towards ultimately-thin multiferroic materials^16,17^. However, multiferroic materials often lose their ferroic properties below a critical size, in particular for ferroelectric materials^18–22^. In ferroelectric nanocapacitors, even in short-circuit, the incomplete screening of the depolarizing field can suppress ferroelectricity^20,23,24^. The critical thickness at which the ferroelectric order disappears has been intensively investigated in ferroelectric thin films^24–29^ as well as multiferroic Fe/BaTiO_3_ multilayers^13^. To track the challenge of critical size in magnetoelectric ultrathin films, a novel concept for seeking alternative magnetic constituents rather than the conventional ferromagnetic metal is needed in stabilizing the polarization in ferroelectric constituent.

Recently, a form of two-dimensional semi-hydrogenated graphene sheet, which is referred to as “graphone”, was found to be a ferromagnetic semiconductor with a small indirect gap^30,31^. Semi-hydrogenation breaks the delocalized ${\rm{\pi }}$ bonding network of graphene, leaving the electrons in the unhydrogenated carbon atoms localized and unpaired, which results in large magnetic moments at these sites. Different from graphene with vacancy, substitution and zigzag edge^32–35^, in which magnetism is inhomogenously distributed and the integrity of the structure is destroyed, graphone not only exhibits homogenously distributed single-layer magnetism but also keeps the integrity of the structure. Therefore, two-atom thick graphone is a possible ferromagnetic constituent to replace conventional metal in ultimately-thin multiferroic heterostructures.

Due to the intriguing coupling of ferroelectric polarization and charge carriers in graphene, graphene-ferroelectric heterostructures (without hydrogenation) have been fabricated as field-effect transistors^36–41^ and flexible transparent electrodes^42^. The influence of polarization on the electronic transport of graphene has been studied for graphene-ferroelectric heterostructures^43–45^. Recently, the electronic and magnetic properties of the interface between graphene and ME multiferroics were investigated by using first principles calculations^46^, yet the screening effect of graphene or graphone on the polarization in ultrathin ferroelectric films is unclear so far. In particular, whether there exists an interfacial MEC between the graphone and ferroelectric perovskite oxide is unknown.

Here, by using first principles calculations, we demonstrate that there is a strong interfacial MEC in the ultrathin heterostructures made of graphone and ferroelectric PbTiO_3_ (PTO), which exhibits an undisclosed physical mechanism and significant improvement over the interfacial MEC of conventional metal/ferroelectric heterostructures. We have conducted a systematic exploration for the graphone/PTO heterostructures with different thicknesses using density-functional theories to provide a detailed insight into the interfacial electronic structures and magnetic properties. We first clarify that not all forms of graphone are ferromagnetic and the ground state is a non-magnetic insulator. We then reveal that a magnetization can emerge at the PTO/graphone interface. This magnetization exhibits strong sensitivity to the direction of ferroelectric polarization, and can even be electrically switched on and off by reversing the polarization of PTO layer, yielding an intriguingly non-magnetic -ferromagnetic (NM-FM) phase transition. As a consequence, a giant interfacial MEC of $7.3\times {10}^{-10}{\rm{G}}\times {\rm{c}}{{\rm{m}}}^{2}/{\rm{V}}$ per unit cell is obtained at the graphone/PTO interface, which largely exceeds the prediction of $2.1\times 1{0}^{-10}{\rm{G}}\times {\rm{c}}{{\rm{m}}}^{2}/{\rm{V}}$ in conventional Fe/BaTiO_3_ multilayers^13^. Furthermore, due to the strong interactions of interfacial atoms and the screening of interfacial charge by graphone, a spontaneous ferroelectric polarization can exist at the ultrathin PbTiO_3_ layer less than one nanometer, indicating the disappearance of critical size for ferroic orders. Our finding of strong magnetoelectric effect in the ultimately thin graphone/ferroelectric heterostructures suggests a novel platform to realize the electric control of magnetism for high density and low-power memory devices.

## Results

### The graphone/PTO/graphone heterostructure

We perform density-functional calculations using the Vienna Ab Initio Simulation Package (VASP)^47,48^ on the electronic, magnetic and structural properties of graphone/PTO/graphone trilayers. PbTiO_3_ has a larger out-of-plane polarization and a smaller ferroelectric critical size than common ferroic materials such as BaTiO_3_ and BiFeO_3_^49–51^. In addition, the MEC of PbTiO_3_-based heterostructures is larger than that of BaTiO_3_ system^52^. Therefore, PbTiO_3_ is selected as the ferroelectric layer in the present study. Here, generalized gradient approximation (GGA) with effective Coulomb-exchange interaction U_eff_ = U − J = 3 eV^53^ imposed for Ti-*d* orbitals is employed. The trilayer structure is modeled as a supercell including 3 × 2 graphene and 2 × 1 PTO unit cells in the (001) plane (Fig. S1 in Supplementary Information). In the [001] direction, various thicknesses of PTO layer are described by the generic formula [Graphone*/*(PbO-TiO_2_)_*m*_-PbO/Graphone], where *m* denotes the number of unit cells of PTO layer. The in-plane sizes of the supercell are constrained at 7.81 Å and 3.905 Å in [100] and [010] directions to simulate the growth on SrTiO_3_ substrate. Such a substrate produces an epitaxial strain on PTO and forces a ferroelectric tetragonal phase with polarization perpendicular to the interface^54^ as generally used to control magnetism at the interface in multiferroic heterostructures.

**Figure 1:**
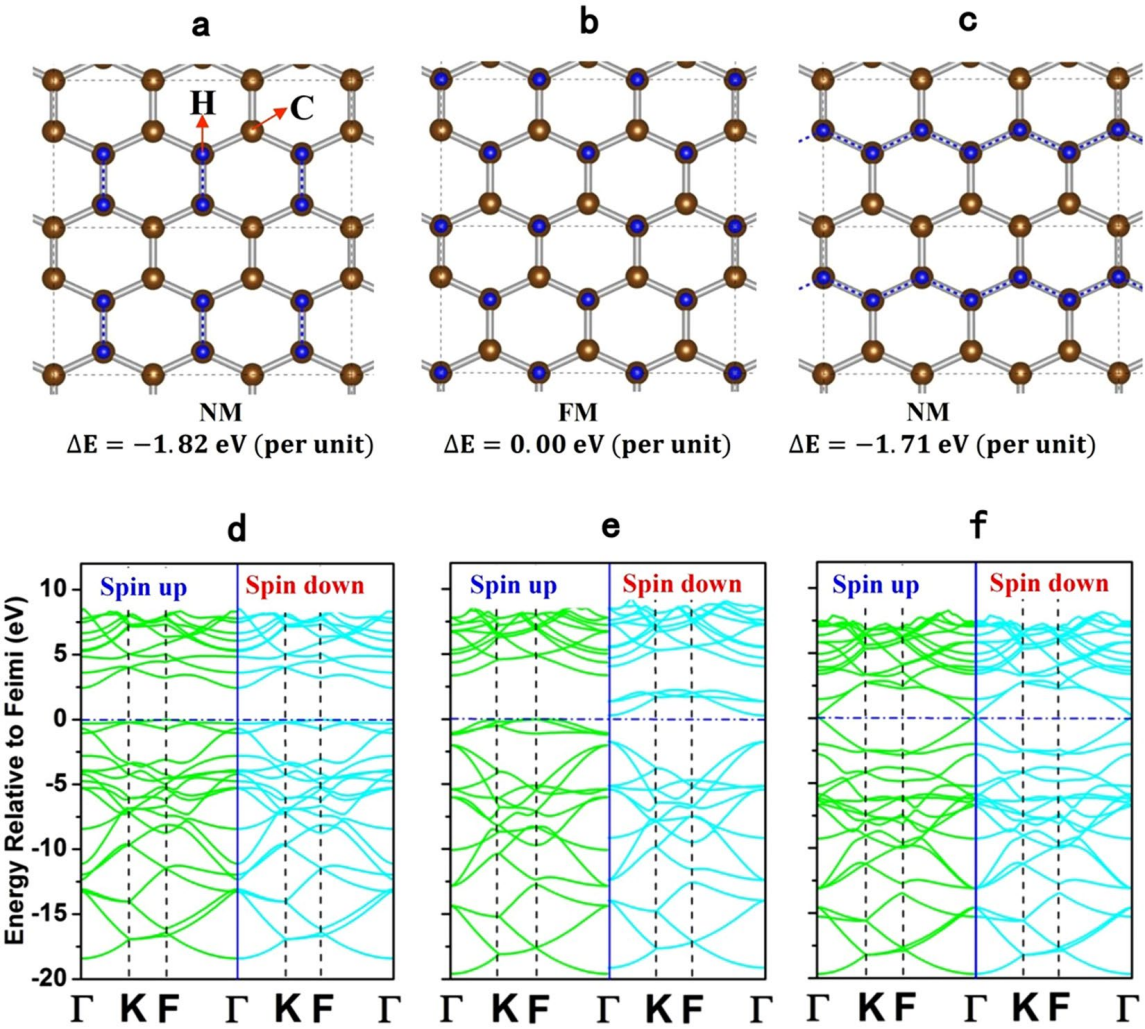
Schematics of the boat (**a**), chair (**b**) and zigzag (**c**) configurations of one-side semihydrogenated graphone. Hydrogen atoms are on the top of the 2D plane and represented by the dark blue spheres. The below corresponds to the energy band structures for the boat (**d**), chair (**e**) and zigzag (**f**) doping graphones, respectively. The energy band structures show that the boat, chair and zigzag doping graphones exhibit the non-magnetic semiconductor, ferromagnetic semiconductor and non-magnetic metallic properties, respectively. The boat doping graphone has the lowest energy per unit cell and is the most stable among three doping graphones.

For the graphone layer, there are three possible semi-hydrogenated configurations, namely the boat doping, chair doping and zigzag doping, as shown in Fig. 1(a–c), respectively. In order to determine the most stable configuration, the energetic, electronic and magnetic properties of three kinds of isolated graphones are first examined. Both spin-polarized and non-polarized calculations are performed to obtain the magnetic ground state. Interestingly, the chair, boat and zigzag doping graphones exhibit the properties of ferromagnetic semiconductor, non-magnetic semiconductor and non-magnetic metal, respectively. For the boat and zigzag graphones, the doping of hydrogen atoms at two consecutive sublattices partially preserve the π-bonding network of graphene and do not produce any unpaired electron, resulting in non-magnetic behaviors and the symmetric band structures for spin up and spin down, as shown in Fig. 1(d,f), respectively. In case of chair doping, the partial saturation of graphene by H-atoms results in breaking of π-bonding network and ferromagnetism appears from the p-electrons associated with unhydrogeneted carbon atoms. The splitting of energy band structures for spin up and spin down in Fig. 1(e) confirms the ferromagnetic and semiconductor nature of chair doping graphone, which is consistent with the previous calculation^31^. It is also found that the energies of boat and zigzag graphones are 1.82 eV and 1.71 eV lower than that of the chair doping graphone, respectively. Based on the energies, the boat doping is the most stable among the three cases, and is selected in the present study to investigate the interfacial MEC of the graphone/PTO/graphone trilayers.

The energy difference between the magnetic and non-magnetic states of the ferroelectric graphone/PTO/graphone trilayers is now examined. It is found that the magnetic state is more energetically favorable than the non-magnetic state, as shown by the energy difference of two states in Table 1, which implies that the magnetic states are stable in the graphone/PTO/graphone trilayers although the isolated PTO layer and boat doping graphone do not possess individually any magnetism.

**Table 1:** The magnetic moments at the interfaces, the difference of magnetic moments between the top and bottom interfaces, the interfacial MEC coefficients, the energy difference between ferromagnetic and non-magnetic states and the average polarization per unit cell in Graphone*/*(PbO - TiO_2_)_m_ - PbO/Graphone trilayers with different thicknesses of m.

	m = 2	m = 4	m = 6
${\mu }_{{\rm{top}}}$ (${\mu }_{B}$/f.u.)	0.98	1.00	1.00
${\mu }_{{\rm{bot}}}$ (${\mu }_{B}$/f.u.)	0.07	0.05	0.05
${\rm{\Delta }}{\mu }_{B}$ (${\mu }_{B}$/f.u.)	0.91	0.95	0.95
${a}_{s}$ (G · cm^2^/V)	7.0	7.3	7.3
E^FM^ - E^NM^ (meV)	−308	−296	−289
P ($\mu C/{{\rm{cm}}}^{2}$)	122	116	114

### Interfacial magnetoelectric effect

**Figure 2:**
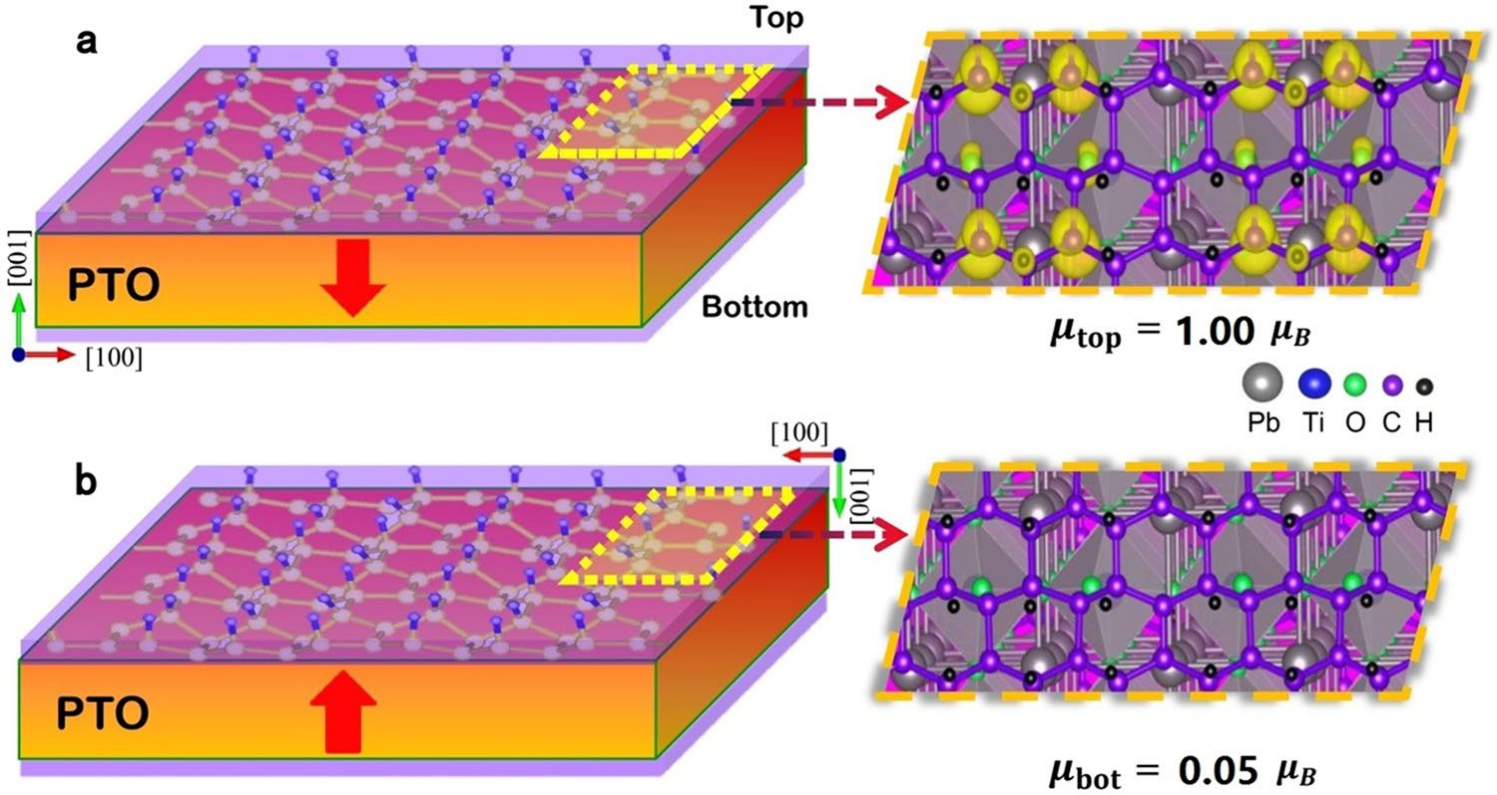
The magnetoelectric effect at the interfaces of graphone/PTO/graphone trilayers with m = 4 and the downward [$00\bar{1}$] polarization (denoted by the arrow) in the PTO layer. (**a**) Strong magnetization appears at the top interface. (**b**) Non-magnetic ground state exhibits at the bottom interface. The right plots show the distribution of the spin charge density (yellow contour), in which the spin density is set to be 0.01*e*Å^−3^. A magnetic to non-magnetic phase transition takes place at the interfaces when the polarization is switched from upwards to downwards by an external electric field.

The magnetism of graphone/PTO/graphone trilayers stems from the interaction of interfacial atoms between graphone and PTO. The symmetry breaking due to polarization in the ferroelectric PTO layer plays a crucial role in changing the atoms interactions and the appearance of magnetism at the interface. When the PTO layer is paraelectric, the structural relaxation is identical at both interfaces as shown by Fig. S1 in Supplementary Information. Atomic interactions are very weak between graphone and PTO and the whole system keeps a non-magnetic state. When the PTO layer is ferroelectric with downward polarization, the polar atomic distortion breaks the symmetry between the top and bottom interfaces, causing changes to the bond configuration at the interfaces. The strong hybridization between C *2p* and O *2p* orbitals at top interface has a significant effect on the local electronic and magnetic structure of graphone layer which is responsible for the appearance of magnetic moments, the origin of which will be discussed later. Figure 2(a) shows the spin charge density at the top interface of the graphone/PTO/graphone trilayers (m = 4) with downward polarization, in which magnetic moments mainly concentrate on the C atoms in the top graphone layer. At the opposite, the spin charge density almost vanishes at the bottom graphone layer as shown in Fig. 2(b). This shows that a magnetic to non-magnetic phase transition will take place at PTO/graphone interfaces when the polarization is switched by an external electric field. Consequently, a novel interfacial magnetoelectric effect is obtained at the graphone/PTO heterostructures.

Next, we check the influence of the thickness of the ferroelectric PTO layer on the interfacial magnetoelectric coupling, which is important for the miniaturization of magnetoelectric devices. Table 1 gives the total magnetic moments at the top and bottom interfaces of the graphone/PTO/graphone trilayers with different thicknesses of PTO layers (1 × 1 PTO unit cell). The total magnetic moments at the top interface are 0.98 ${\mu }_{B}$, 1.00 ${\mu }_{B}$ and 1.00 ${\mu }_{B}$ for m = 2, 4 and 6, respectively, when the polarization is downward. In contrast, the magnetic moments at the bottom interface are negligible, which are 0.07 ${\mu }_{B}$, 0.05 ${\mu }_{B}$and 0.05 ${\mu }_{B}$ for m = 2, 4 and 6, respectively. The difference of magnetic moments of the top and bottom interface ranging from 0.91 ${\mu }_{B}$ to 0.95 ${\mu }_{B}$. The results show that the difference of magnetic moments between the top and bottom interfaces is still sizable when the thickness is less than m = 6, indicating that the large magnetoelectric effect remains at the interface in ultimately-thin graphone/PTO/graphone.

The influence of an electric field *E* on the interface magnetization *M* is usually described in terms of the interfacial MEC coefficient ${\alpha }_{s}$ according to the equation of ${\mu }_{0}{\rm{\Delta }}M={\alpha }_{s}E$. Considering the coercive field of PTO is about 100 kV/cm^51^, the interfacial MEC coefficient ${\alpha }_{s}$ is estimated to be $7.3\times {10}^{-10}{\rm{G}}\cdot {{\rm{cm}}}^{2}/{\rm{V}}$ for the graphone/PTO/graphone trilayers with m = 4, which is more than three times larger than that of the Fe/BTO multiferroic heterostructures^13^. To investigate the influence of different exchange-correlation functionals, the interfacial MEC coefficient is also estimated based on the DFT calculations with PBE and PBEsol functionals. The calculated values are $7.3\times {10}^{-10}{\rm{G}}\cdot {{\rm{cm}}}^{2}/{\rm{V}}$ and $6.5\times {10}^{-10}{\rm{G}}\cdot {{\rm{cm}}}^{2}/{\rm{V}}$, respectively, which implies that the interfacial MEC coefficient is less dependent on the exchange-correlation functional.

### Origin of the magneto-electric coupling

**Figure 3:**
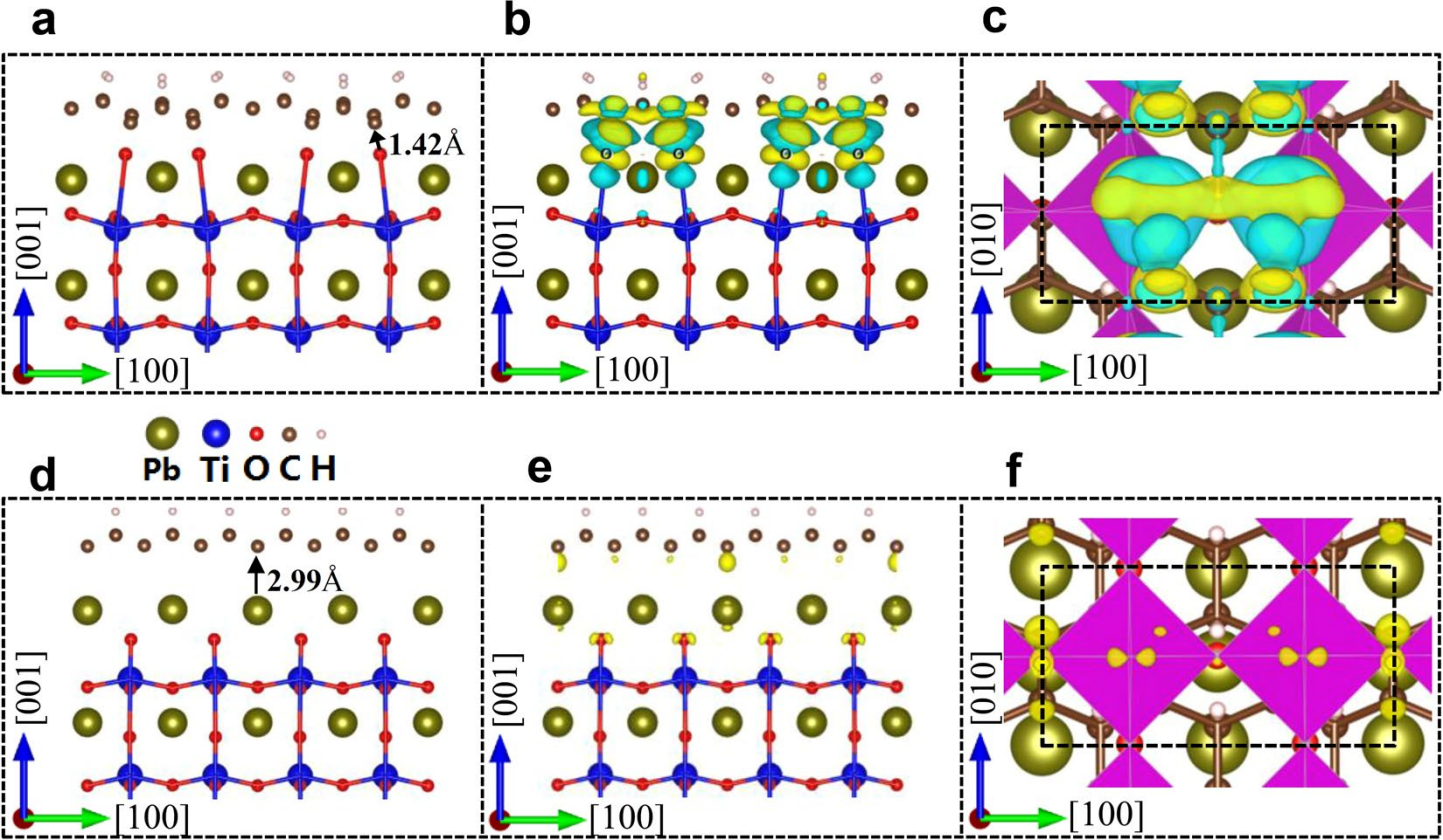
Atomic structure and the charge density difference at the top interface (**a**–**c**) and bottom interface (**d**–**f**) of graphone/PTO/graphone trilayers with m = 4 and the downward [$00\bar{1}$] polarization in the PTO layer. In (**a**) and (**d**), arrows indicate schematically displacements of O and Pb atoms, respectively. (**b**) and (**e**) give the contour plots of charge density difference in the (010) plane; (**c**) and (**f**) are the contour plots of charge density difference in the (001) plane, in which the yellow and green contours denote the gain and loss of electrons, respectively. The contour value is set to be 0.004 *e*Å^−3^.

The predicted giant MEC at graphone/PTO interface can be understood from the change in atomic interactions and charge density difference. The polarization induces asymmetric atomic displacements across the interfaces and results in an orbital mixing. Figure 3(a) shows that downward polarization brings the interfacial oxygen atoms close to the graphone layer at top interface. The nearest distance between C and O atoms at top interface is only 1.42 Å, which facilitates a stronger interaction between C (cation) and O (anion). The strong atomic interaction leads to a strong charge transfer as shown by charge density difference in Fig. 3(b,c), which results in nonzero spin charge density mainly localized on the interfacial C atoms in Fig. 2(a). At the opposite, the oxygen atoms at the bottom interface are far from the graphone layer. There is a C-Pb (cation-cation) interaction but it is weak and yielding a small charge transfer at the bottom interface as revealed by the charge density difference in Fig. 3(e,f).

**Figure 4:**
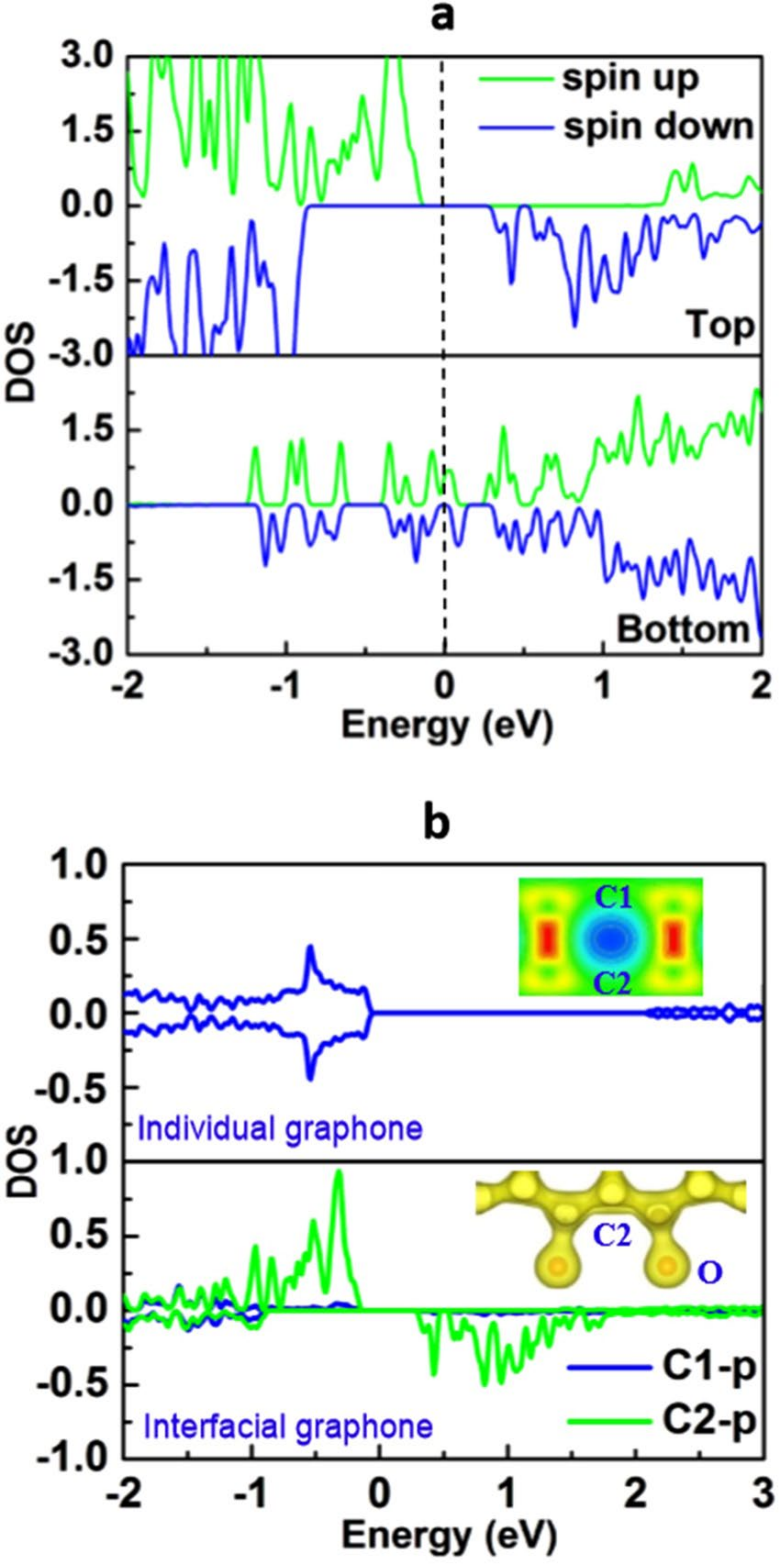
(**a**) Orbital-resolved density of states for interfacial atoms at the top and bottom interfaces of graphone/PTO/graphone trilayers with m = 4 and the downward polarization in the PTO layer. (**b**) Projected DOS of C-2p orbit of two neighboring un-doped carbon atoms in the individual graphone and the graphone at the top interface. The insets show the structural and electronic configuration of carbon and oxygen atoms.

The MEC in graphone/PTO can also be qualitatively estimated from the density of state presented in Fig. 4. It is found that the top interface with downward polarization forms highly spin-polarized states close to the Fermi level as compared to that of bottom interface (Fig. 4(a)), suggesting that polarization in the heterostructure is associated with the depletion and accumulation of the charge density at top and bottom interfaces, respectively. To give a further insight into the origin of a sizable magnetization at the interface, projected density of states (PDOS) of two nearest-neighbor carbon atoms in isolated boat graphone and interfacial graphone are shown in Fig. 4(b). For isolated graphone, the unpaired electron in two C atoms form the π-bonding and there is no spin splitting. For graphone at the heterostructure interface, due to the strong interaction between one of the two C atoms with the interfacial oxygen atom, electron transfers from the carbon to the oxygen atom and the local π-bonding network is broken. As a result, the unpaired electron in another carbon atom forms the spin-polarized states just below the Fermi with a high spin moment of 0.64 ${\mu }_{B}$. Thus, the difference in the spin moments at the top and bottom interfaces results in a high MEC for the graphone/PTO heterostrucuture.

Although the graphone with boat doping exhibits the lowest energy, the energy difference between the zigzag and boat graphones is quite small, indicating that the zigzag and boat graphones may coexist in practical situation. To verify the generality of above magneto-electric coupling, the existence of MEC in the graphone/PTO heterostrucuture with zigzag doping is also examined. Fig. S2 shows the spin charge density of zigzag graphone/PTO heterostructures with m = 4. Similar to the above boat doping, a sizable magnetization is found at the top graphone layer with zigzag doping due to the strong interactions between interfacial carbon and oxygen atoms, and the bottom layer remains non-magnetic. The calculated MEC coefficient ${\alpha }_{s}$ with zigzag doping is $7.3\times {10}^{-10}{\rm{G}}\cdot {{\rm{cm}}}^{2}/{\rm{V}}$, which is the same as that of boat doping. These results highlight that electric field controlled magnetism is generic no matter in the boat type or less stable zigzag type, which is important for realizing the magnetoelectric effect in experiments because it may be difficult to precisely control the doping position of hydrogen atoms.

### Absence of ferroelectric critical size

**Figure 5:**
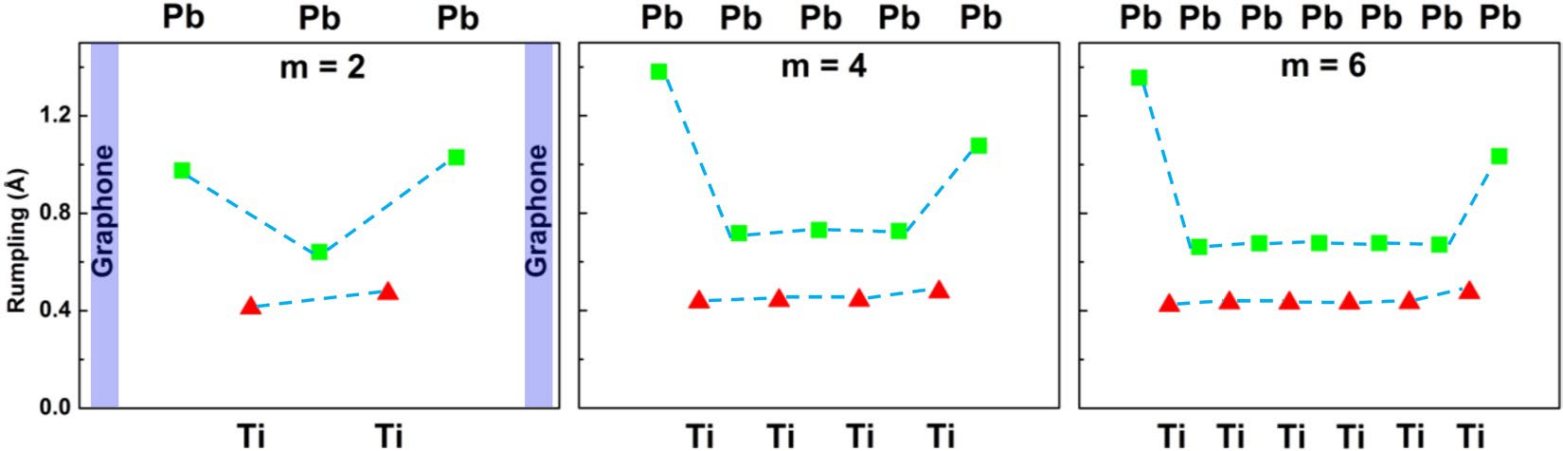
Atom layer rumpling (the relative displacement of cation-oxygen) profiles obtained from fully relaxed graphone/PTO/ graphone trilayers, (**a**–**c**) show different thicknesses of ferroelectric layers. The rumpling does not decrease when the thickness of PTO layer decreases, which makes the polarization stable at a thickness of two-unit cell.

As shown above, the presence of ferroelectric polarization in PTO layer causes the difference of magnetic moments at the top and bottom interfaces of graphone/PTO/graphone trilayers, which results from the change in the strength of bonding between C and O atoms. Therefore, the stability of ferroelectric distortion in PTO layer is crucial for the existence of interfacial MEC of graphone/PTO/graphone heterostructure. The polarizations in the PTO layer with different thicknesses are calculated and listed in Table 1. It is found that the polarization not only can survive in ultrathin layers down to 2 unit-cells as observed in experiments^27,28^ but also have a larger value than bulk polarization, indicating the enhancement of the polarization at the interface in a way similar to what was predicted at interface between AO-terminated perovskites and simple metals^24^. This polarization enhancement is due to the strong atomic interaction at the interface. The atomic interactions at both top and bottom interfaces induces a larger Pb-O relative displacements in the interface layers than in the middle layers as shown by the profiles of atom rumpling (i.e. the cation-oxygen relative displacements within each atomic layer) in Fig. 5. Furthermore, the rumpling does not decrease when the thickness of PTO layer decreases, which makes the polarization stable at a very small size. To further support the validity and effectiveness of the GGA + U method in the prediction of critical thickness, PBEsol functional^55^ for solids and surfaces is also employed to relax the structure and to calculate the rumpling as shown in Fig. S3 in the Supplementary Information. It is clear that a spontaneous polarization can be stabilized in two-unit cell thick layer, although the rumpling amplitude is slightly smaller than at the GGA + U level.

The absence of ferroelectric critical size in graphone/PTO/graphone trilayers may also be understood from the charge transfer at the top and bottom interfaces. At the top interface, there are more electrons lost than gained when the polarization is downward as shown in Fig. 3(c), resulting in net positive charges that screen the polarization induced negative charges. The situation is reversed for bottom interface, in which more electrons gained to screen the polarization induced positive charges as shown by Fig. 3(d). The charge transfer provides the partial screening at both interfaces, which decreases the depolarization field and thus stabilizes ferroelectric distortion in the ultrathin PTO layer. In addition, the change of PTO thickness has not too much influence on the MEC of graphone/PTO/graphone trilayers, as shown by the interfacial MEC coefficient ${\alpha }_{s}$ for different thicknesses in Table 1. The ultimately thin graphone/PTO/graphone trilayers not only possess stable polarization but also exhibit strong magnetoelectric coupling, which suggests an ultrathin multiferroic heterostructures for future ultrahigh spintronic memories.

## Discussion

In summary, we have conducted a systematic exploration for graphone/PTO/graphone trilayers of different thicknesses which together revealed and provided a detailed insight into a sizable interface MEC. Several interesting and practically useful properties are predicted. Firstly, we demonstrate that the magnetization emerging in the graphone layer exhibits strong sensitivity to the direction of the ferroelectric polarization, and can even be electrically switched on and off by reversing the polarization, yielding a novel non-magnetic-ferromagnetic (NM-FM) phase transition. As a consequence, a giant interfacial MEC of $7.3\times {10}^{-10}{\rm{G}}\times {\rm{c}}{{\rm{m}}}^{2}/{\rm{V}}$ is obtained at the interface of the trilayers. In addition to the strong MEC at the interface between the graphone and ferroelectric PbTiO_3_, an undisclosed physical mechanism, in which the change of carbon-oxygen bonding at the interface plays an important role, is predicted for the MEC based on the detailed analysis of electronic structures.

To track the challenge of critical size in magnetoelectric ultrathin films, a novel graphone magnetic constituents rather than the conventional ferromagnetic metal is proposed to stabilize the spontaneous polarization in ultrathin ferroelectric constituent. Due to the strong carbon-oxygen bonding and the screening of interfacial charge by graphone, a spontaneous polarization can exist at the ultrathin PbTiO_3_ layer less than one nanometer, indicating the disappearance of critical size for ferroic orders. The proposed graphone/PTO/graphone multiferroic heterostructure exhibits significant improvements over the conventional Fe/BTO multiferroic heterostructure: (i) The MEC of graphone/PTO/graphone is more than three times in magnitude larger than that of Fe/BaTiO_3_; (ii) The single layer nature of graphone instead of thick metal electrode makes the multiferroic heterostructure ultimately thin, which may increase the density of spintronic memories; (iii) The voltage to switch the polarization is greatly reduced due to the ultrathin nature of ferroelectric layer in the multiferroic heterostructure. Our finding of strong magnetoelectric effect in the ultimately thin graphone/ferroelectric films opens a new perspective to the electric control of magnetism for high density and low-power memory devices.

## Methods

First principles calculations are performed based on the density functional theory (DFT) using the projector-augmented wave (PAW) method, which are implemented in the Vienna *ab initio* simulation package (VASP)^47,48^. GGA + U method^53^ with effective Coulomb-exchange interaction U_eff_ = U − J = 3 eV for Ti-*d* orbitals is employed, in which U is the Coulomb interaction parameters and J is the exchange interaction parameters. The GGA + U method could better describe the electronic states than GGA and LDA. Careful test calculations show that a relatively high plane-wave cutoff energy of 500 $\mathrm{eV}$ and a 7 × 3 × 1 Monkhorst-Pack *k-*point mesh^56^ give well-converged results and are enough to accurately describe the electronic properties. A vacuum region of about 20 Å in the direction normal to graphene plane was employed to avoid interaction between adjacent layers. During the structural optimizations, the supperlattices are fixed, while relaxation takes place in the internal coordinates of atoms. The atomic structures are fully relaxed using the conjugate gradient method until the Hellmann-Feynman forces on each atom are less than 0.01 ${\rm{e}}{\rm{V}}/\backslash \AA$. The polarization (per unit cell) is estimated from the Born effective charge method as $\,P=\frac{e}{{\Omega }_{c}}\sum _{j}{w}_{j}{Z}_{j}^{\ast }\delta {u}_{j}$, in which *P* denotes the spontaneous polarization along the [001] direction. ${\Omega }_{c}$ and $\delta {u}_{j}$ are the primitive cell volume and the displacements of atom relative to the centrosymmetric structure, respectively. Index $j$ covers all atoms in the unit cell, weights ${w}_{j}$ are set to1/8 for Pb, 1 for Ti, and 1/2 for O, which represents the number of unit cells that share the atom and $\,{Z}_{j}^{\ast }$ is the Born effective charge tensor calculated from density functional perturbation theory^57^.

## Electronic supplementary material

Supplementary Information
