# Supplementary material for: Giant magnetoelectric effect at the graphone/ferroelectric interface
Source: Sci Rep. 2018 Aug 20;8:12448. doi: 10.1038/s41598-018-30010-x (PMC6102284; doi:10.1038/s41598-018-30010-x)
Supplement: Supplementary file 1 — Supplementary Information [file 41598_2018_30010_MOESM1_ESM.docx]

Supplementary Information for

**Giant magnetoelectric effect at the graphone/ferroelectric interface**

Jie Wang^1^[[1]](#footnote-1)^*^, Yajun Zhang^1, 2^, M.P.K. Sahoo^1^, Takahiro Shimada^3^, Takayuki Kitamura^3^, Philippe Ghosez^2^ and Tong-Yi Zhang^4^

*^1^Department of Engineering Mechanics & Key Laboratory of Soft Machines and Smart Devices of Zhejiang Province, Zhejiang University, 38 Zheda Road, Hangzhou 310007, China*

*^2^Theoretical Materials Physics, Q-MAT, CESAM, University of Liège, B-4000 Liège, Belgium*

*^3^Department of Mechanical Engineering and Science, Kyoto University,*

*Nishikyo-ku, Kyoto 615-8540, Japan*

*^4^Shanghai University Materials Genome Institute and Shanghai Materials Genome Institute,*

*Shanghai University, 99 Shangda Road, Shanghai 200444, China*


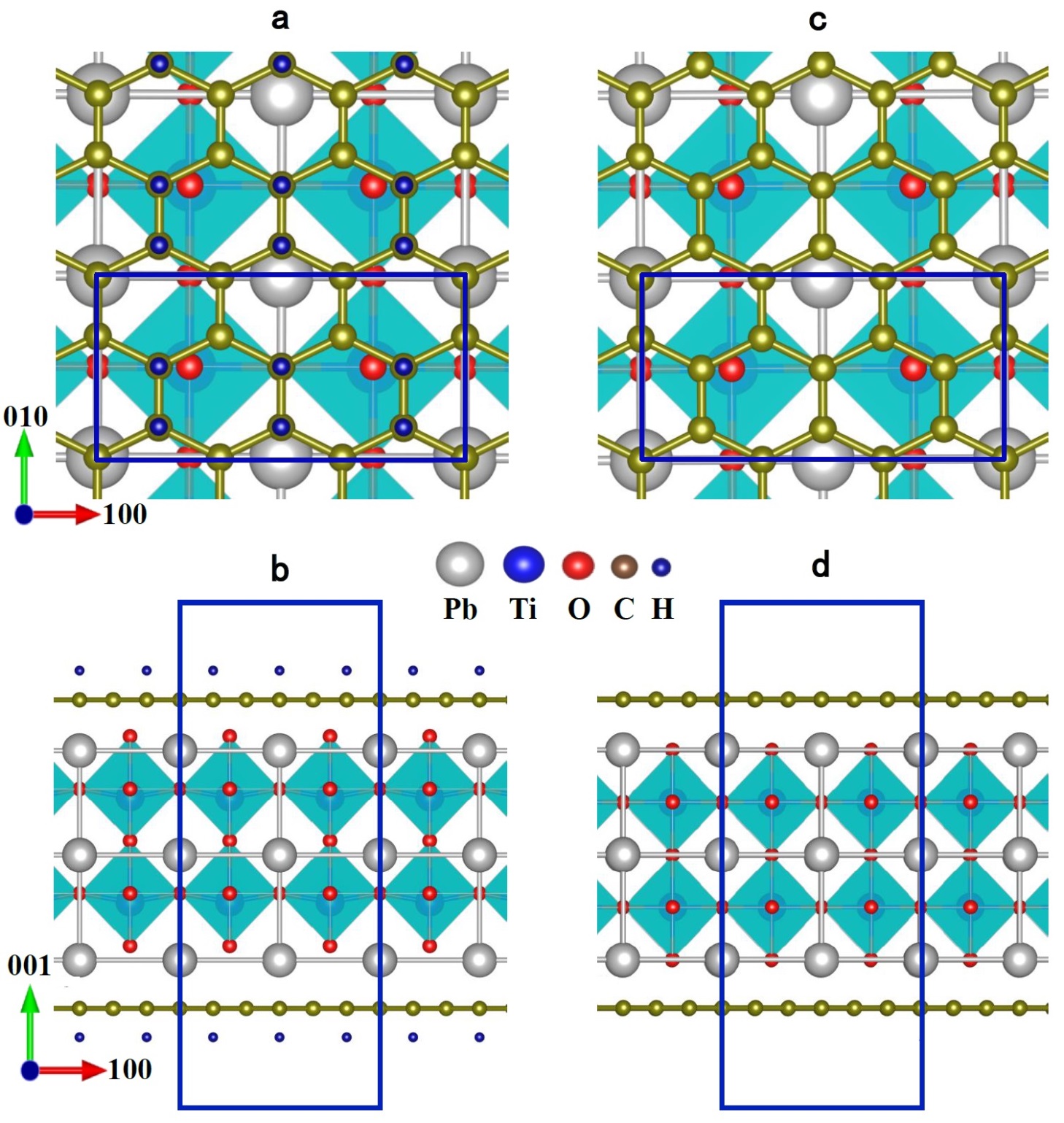


**Figure S1** (a) Top view and (b) side view of graphone/PTO/graphone trilayers with m= 2, in which the downwards [$00\bar{1}$] polarization stably exists in the PTO layer due to the screening effect of graphone to polarization; (c) Top view and (d) side view of graphene and PTO heterostructure, in which ferroelectric polarization disappears in PTO layer, implying the primitive graphene without hydrogenation has no screening effect to polarization. The blue rectangle frames denote the supercells used in the calculations.


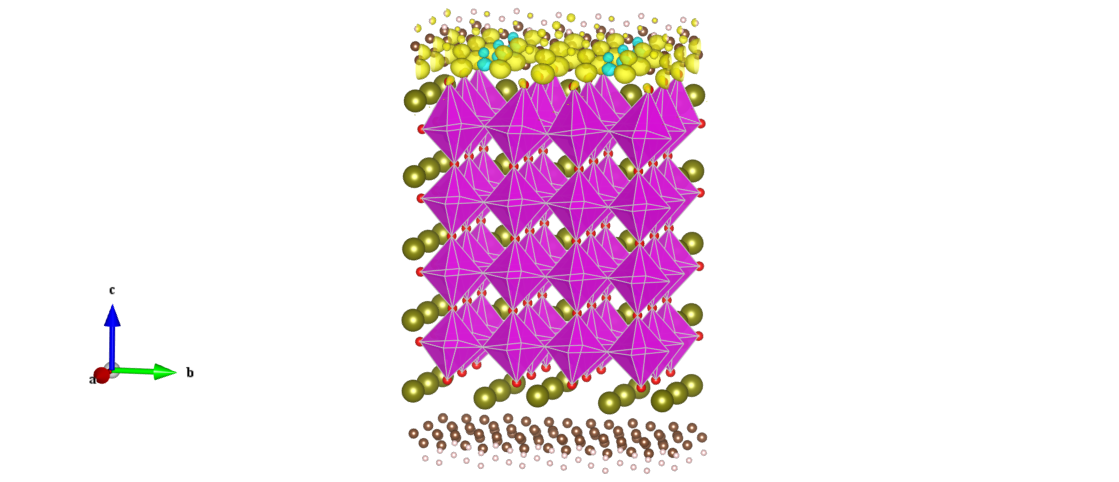


P

**Figure S2** Spin charge density of zigzag graphone/PTO heterostructures with four layers PTO and downwards polarization. A sizable magnetization appears at the top graphone layer due to the strong interactions between interfacial carbon and oxygen atoms, whereas the bottom layer remains non-magnetic.


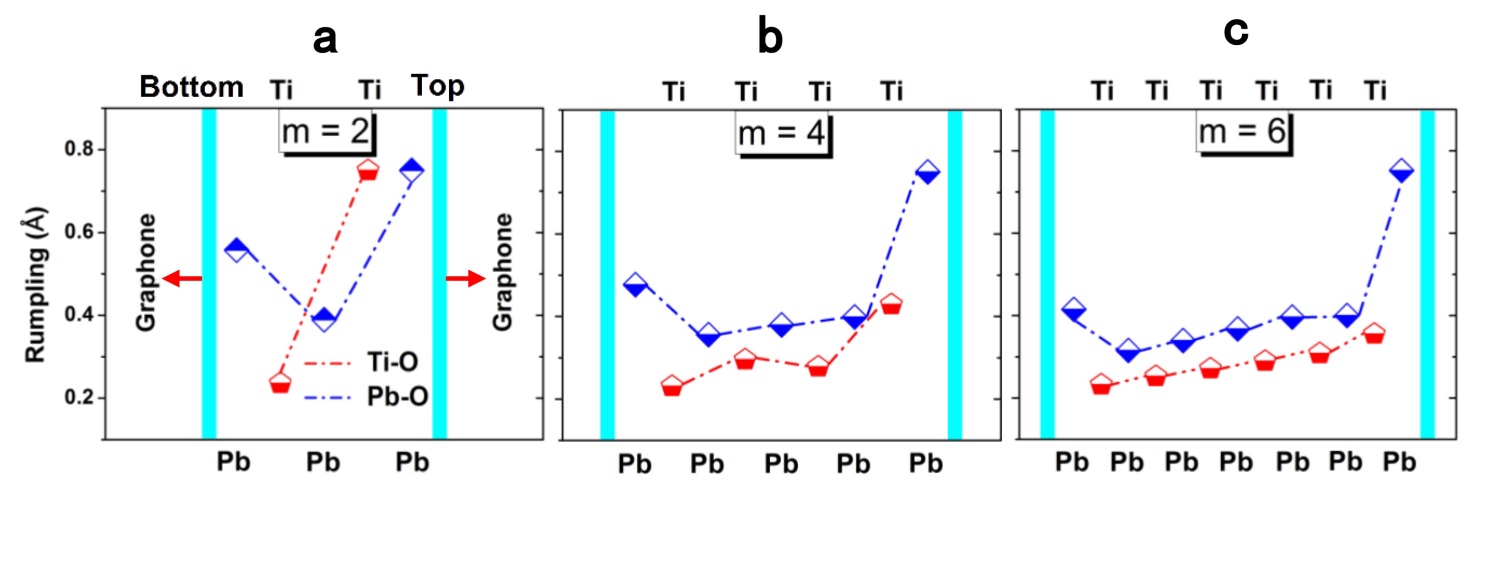

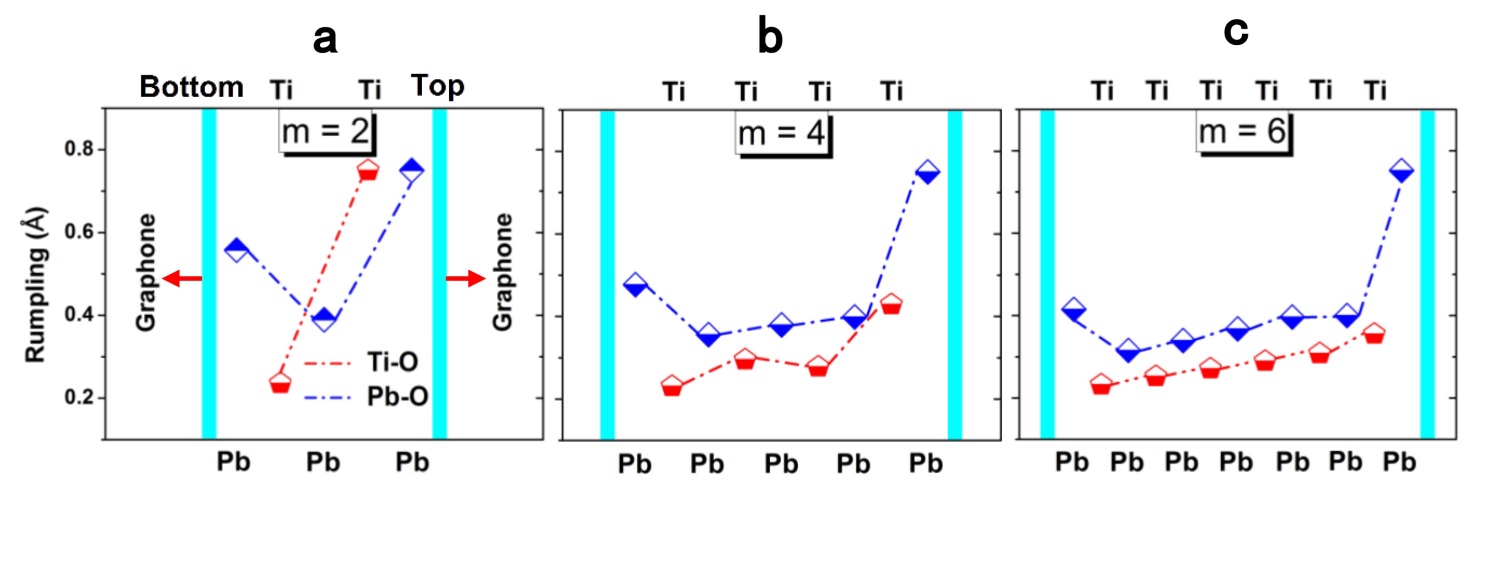

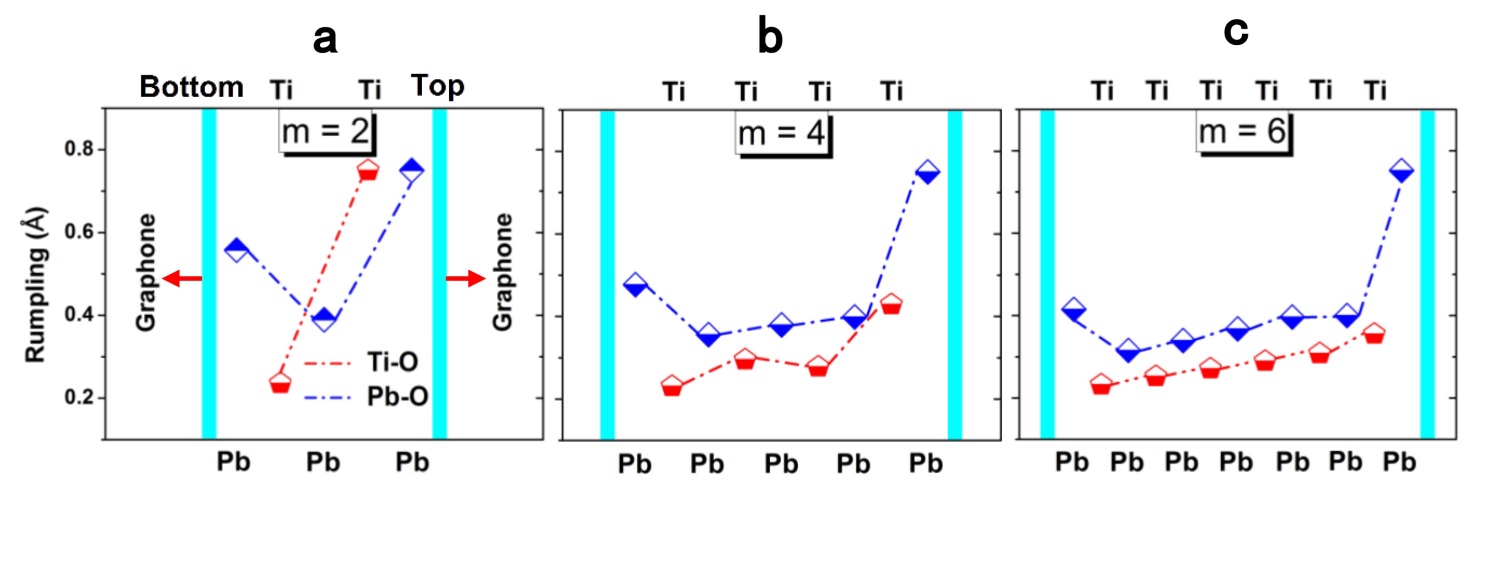


**Figure S3** Atom layer rumpling profiles obtained from fully relaxed graphone/PTO/ graphone trilayers from PBEsol functional, (a)–(c) show different thicknesses of ferroelectric layers. The polarization can be stable in the PTO layer with the thickness of two-unit cell.

1. * *E-mail:jw@zju.edu.cn* [↑](#footnote-ref-1)
